# Effect of Global Longitudinal Strain at Discharge Period on Predicting Cardiac Defibrillator Implantation in STEMİ Patients with Impaired Left Ventricle Systolic Functions

**DOI:** 10.3390/medicina61030545

**Published:** 2025-03-20

**Authors:** Ömer Ferudun Akkuş, Muhammet Gürdoğan

**Affiliations:** Department of Cardiology, School of Medicine, Trakya University, 22030 Edirne, Turkey; drmgurdogan@gmail.com

**Keywords:** global longitudinal strain, heart failure, implantable cardioverter defibrillator, ST-segment elevation myocardial infarction, mortality

## Abstract

*Background and Objectives*: Contemporary studies have shown that low ejection fraction (EF) is a significant predictor of sudden cardiac death in ischemic heart failure. However, relying solely on EF and waiting 90 days for ICD implantation is inadequate for preventing sudden death in STEMI patients. *Materials and Methods*: This study aims to explore if left ventricular global longitudinal strain (GLS) measured at discharge can predict EF < 35% at the third-month follow-up in STEMI patients with impaired systolic function (EF < 35%). The study involved 69 patients diagnosed with STEMI. The results from 29 patients with EF ≤ 35% and 40 patients with EF between 36 and 49% were compared. Echocardiographic images were recorded, and the LV GLS value was measured. *Results*: In both univariate and multivariate regression analyses, LV GLS at discharge was the only independent predictor of EF ≤ 35% after three months. An LV GLS value below 9.55% at discharge predicted an EF below 35% at 90 days, with 75% sensitivity and 76.5% specificity (AUC = 0.814, *p* = 0.005). *Conclusions*: Current guidelines recommend waiting three months before ICD implantation in STEMI patients with EF ≤ 35%, but this study suggests that low GLS can help identify high-risk patients earlier, potentially reducing the waiting period for ICD implantation.

## 1. Introduction

Nearly half of patients with ischemic heart failure following STEMI (ST-Elevation Myocardial Infarction) succumb within the first five years post-index event, primarily due to progressive pump failure or arrhythmic complications. Contemporary clinical studies on patients with ischemic heart failure have demonstrated that low ejection fraction is one of the most significant predictors of sudden cardiac death [1,2]. Therefore, current treatment guidelines recommend the use of implantable cardioverter defibrillator (ICD) therapy for the primary prevention of sudden death in selected patient groups who develop heart failure due to ischemic heart disease [3]. According to evidence-based guidelines that inform our current treatment practices, ICD implantation for the primary prevention of sudden death is recommended in ischemic heart failure patients based on assessments conducted in the third month post-discharge. Specifically, patients with an ejection fraction (EF) ≤ 35% and functional capacity of class 2 or 3 according to the New York Heart Association (NYHA) classification, as well as those with an EF ≤ 30% and NYHA class 1 functional capacity, are advised to receive ICD therapy for primary prevention [4]. In fact, the guidelines also recommend the use of a wearable cardioverter defibrillator (WCD) with a Class 2B indication in selected patients after myocardial infarction [3]. The guidelines recommend measuring EF values at an optimal time of at least 40 days post-STEMI and 3 months following revascularization through echocardiographic evaluation. This timing is suggested primarily because in some patients with an early EF measurement of ≤35%, optimal medical treatment after discharge can reverse adverse left ventricular remodeling and improve EF values, thereby eliminating the indication for ICD implantation in these patients [5,6,7,8]. Although guidelines emphasize an EF threshold of ≤35% for the prevention of sudden death in heart failure patients, studies report that 70–80% of patients experiencing out-of-hospital cardiac arrest have an EF > 35%. Therefore, relying solely on the EF criterion and waiting 90 days may not be sufficient to prevent sudden death in patients who develop ischemic heart failure post-STEMI. This highlights the need for additional diagnostic methods to improve risk assessment in these patients [9]. In the literature, evidence suggests that global longitudinal strain (GLS) measurement is superior to EF in assessing left ventricular systolic function, as well as in predicting mortality and arrhythmias following STEMI [10,11]. As an accessible and repeatable echocardiographic tool, we believe that left ventricular GLS may be valuable in identifying high-risk patients for the prevention of sudden cardiac death, in addition to EF, when determining the need for ICD implantation. This study aims to investigate whether the left ventricular GLS value measured at discharge in STEMI patients with impaired left ventricular systolic function (EF ≤ 35%) who were discharged on optimal medical therapy can predict low EF at the 3-month follow-up.

## 2. Method

This study was designed as a prospective cohort study.

### 2.1. Study Population

The study was conducted on patients admitted to the coronary intensive care unit of a Trakya University hospital with a diagnosis of STEMI and who underwent primary percutaneous coronary intervention between 1 March 2021 and 1 March 2022. The study was conducted in accordance with the Declaration of Helsinki, and ethical approval was obtained prior to initiation (TUTF-BAEK-2021/96). The study initially included 113 patients who were diagnosed with STEMI, underwent primary percutaneous intervention, consented to participate in the research, and had suitable echocardiographic imaging for strain analysis. A total of 44 patients were excluded from the analysis, including 3 patients with a history of previous MI and an EF value below 50% according to hospital records, as well as 41 patients whose echocardiographic evaluation at discharge revealed an (EF) of ≥50%. The remaining 69 patients were classified as having impaired left ventricular systolic function in line with current guidelines and were divided into two groups based on EF values. Group 1 included patients with an EF ≤ 35%, while Group 2 comprised those with an EF of 36–49%. Sociodemographic, clinical, angiographic, and echocardiographic data were recorded for both groups. EF values were obtained from images taken at discharge and at the 3-month follow-up, while left ventricular (GLS) values were recorded based on the images taken at discharge.

### 2.2. Study Flowcharts

**Scheme 1:**
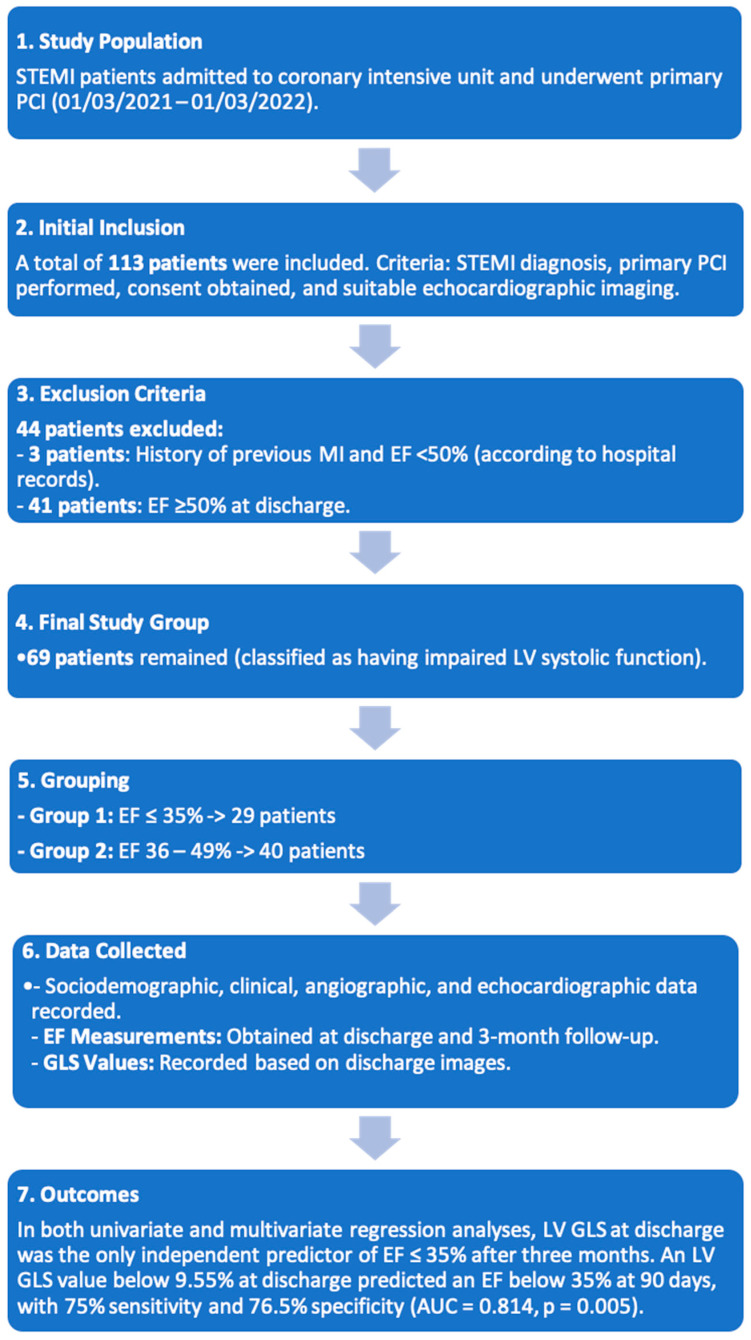
Study Flowcharts.

The study’s sample size, inclusion and exclusion criteria, methodology, and results are shown in the flow chart below (Scheme 1).

### 2.3. Echocardiographic Evaluation

#### 2.3.1. Conventional Echocardiographic Measurements

Echocardiographic images were recorded using Vivid S70 systems (GE Healthcare, Horten, Norway) and transferred to EchoPAC software (application software version: 6.1.3). All measurements were performed in accordance with current echocardiography guidelines [12]. Apical 4-chamber, apical 3-chamber, and apical 2-chamber images were obtained. In addition to these images, tissue Doppler measurements, M-mode measurements, and PW (pulse wave) Doppler measurements were conducted. Left ventricular ejection fraction was calculated using the modified Simpson method in a biplane approach. In the parasternal long-axis view, M-mode was employed along the same axis to measure end-systolic and end-diastolic diameters, wall thicknesses, and left atrial diameter. In the apical 4-chamber view, right atrial and right ventricular diameters were measured. Using PW Doppler in the apical 4-chamber view, the early diastolic filling velocity (E wave), peak filling velocity during atrial systole (A wave), and E/A ratios were calculated. E′ values were measured using tissue Doppler and PW Doppler. The average of the lateral and septal E′ values was taken, and E/e′ ratios were recorded.

#### 2.3.2. Two-Dimensional Strain Echocardiographic Analyses

**Figure 1:**
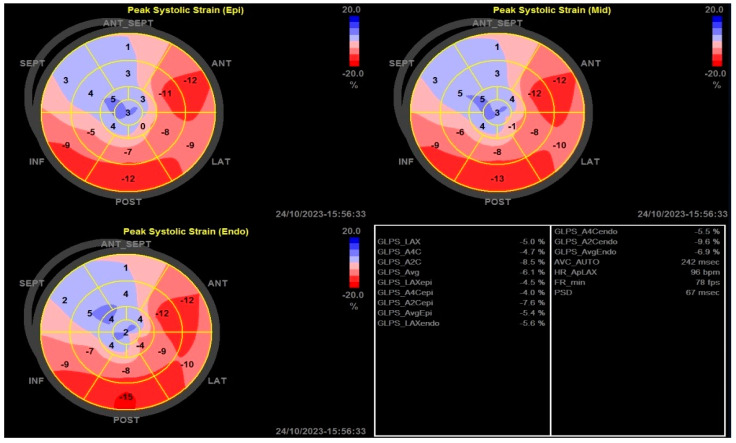
Bullseye graphics of a patient with severely impaired left ventricular systolic function, with a GLS value of −6.1%. Two-dimensional echocardiography reveals dyskinesia in the anteroapical segments of the left ventricle.

To perform strain analysis measurements, all images were transferred to the EchoPAC analysis software. Strain analyses were calculated over three cardiac cycles using apical 4-chamber, apical 3-chamber, and apical 2-chamber images at a frame rate of 50–80 frames per second. All strain analyses were conducted according to current measurement guidelines. In the measurements, one point on the apex and a point from each corner of the mitral annulus were identified. The software automatically traced the myocardial borders. Where necessary, manual adjustments were made to the point tracing to optimize the measurements for accuracy [13]. After manual adjustments, the strain measurements were automatically calculated by the software. In the apical 3-chamber view, the point of aortic valve closure was identified as the end of systole. After processing the images from the three different views, a 17-segment bullseye model was created in the software (Figure 1). The GLS values were automatically calculated by the software as percentage values. All regional longitudinal strains (RLSs) were computed based on the average peak strain values of all segments, categorized according to the perfusion territories of the three major coronary arteries, using the 17-segment model for all layers [14].

### 2.4. Statistical Analysis

Statistical analyses were performed using SPSS version 20.0 (IBM Co., Armonk, NY, USA). Data are expressed as mean ± standard deviation (SD), median (interquartile range), or count (%). The normality assumption was tested using the Shapiro–Wilk test. Categorical variables between groups were compared using the Pearson Chi-Square test. Differences between the patient group and the control group were assessed with an independent samples *t*-test for parametric variables and the Mann–Whitney U test for non-parametric variables. For patients with EF ≤ 35%, stepwise univariate and multivariate logistic regression analyses were employed. Area under the curve values and cutoff points were obtained using ROC analysis. A *p*-value of <0.05 was considered statistically significant.

## 3. Results

A total of 69 patients with impaired left ventricular systolic function were divided into two groups based on ejection fraction (EF) values: Group 1 (patients with EF ≤ 35%) and Group 2 (patients with EF 36–49%). Group 1 consisted of 29 patients (20.6% female), while Group 2 included 40 patients (63.3% female). The average age of Group 1 was 60.2 ± 9.5 years, while the average age of Group 2 was 54.7 ± 11 years (*p* = 0.03). A higher number of patients with a history of hyperlipidemia was observed in Group 1 (*p* = 0.001). In Group 1 patients, the GFR (glomerular filtration rate) value was significantly lower (*p* = 0.03), peak troponin levels were significantly higher (*p* = 0.05), and the number of affected vessels was greater (*p* = 0.001). In Group 1 patients, a higher average age, a greater number of affected vessels, and higher peak troponin levels compared to Group 2 may explain the lower EF observed in this group. There were no statistically significant differences between the two groups regarding the localization of myocardial infarction on ECG, door-to-balloon time, the coronary artery associated with the infarction, or other parameters. A comparison of socio-demographic characteristics, cardiovascular risk factors, and baseline hemodynamic and laboratory data of both groups is presented in Table 1, while a comparison of angiographic findings, discharge medications, and three-month follow-up outcomes is provided in Table 2. There was no significant difference between the groups in terms of recurrent hospitalizations, recurrent MI, and mortality during the three-month follow-up period. However, due to a proportionally higher number of diseased vessels, the number of repeat interventions was found to be significantly higher (*p* < 0.001).

**Figure 2:**
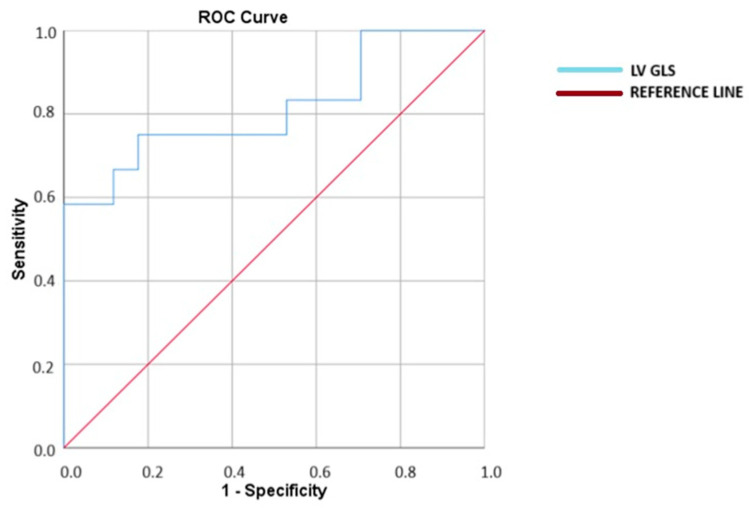
Predictive impact of GLS at discharge on EF ≤ 35% at the 3-month follow-up (GLS AUC 0.814 (95% CI: 0.642–0.986), *p* = 0.005, sensitivity: 75%, specificity: 76.5%) (ROC analysis).

When comparing echocardiographic data between the two groups, a statistically significant difference was found in EF values at discharge and at the 3-month follow-up after discharge (*p* < 0.001). At discharge, the average LV GLS value for Group 1 patients was −8.9%, while the average LV GLS value for Group 2 patients was −15.1%. This difference was statistically significant (*p* < 0.001). The left ventricular diastolic and end-systolic diameters of Group 1 patients were significantly larger compared to those in Group 2 (*p* < 0.001). Additionally, the left atrial diameter and E/e′ ratio in Group 1 patients were significantly higher than those in Group 2 (*p* < 0.001) (Table 3). In the univariate and multivariate regression analyses conducted to identify factors predicting EF ≤ 35% at 3 months post-STEMI, only the LV GLS value measured at discharge was found to be an independent predictor (*p* = 0.042) (Table 4). It was determined that an LV GLS value below 9.55% at discharge indicated that EF would remain below 35% by day 90 post-discharge, with a sensitivity of 75% and specificity of 76.5% (AUC = 0.814, *p* = 0.005). The ROC curves and analysis results are shown in Figure 2.

## 4. Discussion

The key findings of this study can be summarized as follows: (1) In patients who developed left ventricular systolic dysfunction following STEMI, LV GLS analysis performed at discharge may serve as a predictor of LV EF at the 3-month follow-up. (2) In patients with an LV GLS value below 9.55% at discharge, LV EF remains ≤35% at the third month despite optimal medical treatment. (3) LV GLS obtained from speckle tracking echocardiography can be used as a complementary method to LVEF for assessing LV systolic function in making treatment decisions. LVEF is currently the most validated and widely used echocardiographic marker, serving as a selection criterion for medical or device therapies based on evidence. However, GLS assessment obtained from speckle tracking echocardiography (STE) offers a precise and feasible method that overcomes many limitations of LVEF, including reproducibility issues in serial testing and the detection of LV dysfunction in pathologically remodeled hearts [15]. Multicenter post-infarction studies have demonstrated a close relationship between the degree of left ventricular systolic dysfunction and the development of cardiovascular and all-cause mortality [16]. A study conducted three decades ago demonstrated that, over an average follow-up period of 12 years, survival was 21% in patients with an LVEF ≤ 35%, 54% in those with an LVEF between 36% and 49%, and 73% in patients with a normal ejection fraction [17]. Despite the remarkable advancements in diagnostic and therapeutic fields today, studies continue to show a strong association between low ejection fraction and mortality [18]. In patients with impaired LV systolic function, a linear relationship has been reported between GLS and LVEF, with a GLS value of 11% or 12% corresponding to an LVEF of 35% [19]. The greatest advantage of GLS over LVEF is reported to be its ability to detect subclinical myocardial dysfunction in the early stages, before any decrease in LVEF [16]. Currently, LVEF is the primary criterion used for the implantation of cardiac defibrillators for primary prevention in patients with heart failure. However, the prognostic value of EF remains a topic of debate. Studies have reported that strain imaging may serve as a better prognostic factor independent of LVEF [20]. In the literature, a study comparing 70 patients with ischemic or non-ischemic cardiomyopathy who had a cardiac defibrillator implanted and had LVEF ≤ 40% found a significant difference in GLS values between those who experienced arrhythmic events and those who did not over an average follow-up period of 1.8 ± 0.6 years. In patients who experienced arrhythmic events, the GLS value was −6.97 ± 3.06, while in those who did not, the GLS value was −11.82 ± 4.25. The study reported that a GLS value below −10% predicted the occurrence of an arrhythmic event with 90% specificity and 72.2% sensitivity, indicating that GLS is superior to LVEF in predicting ventricular arrhythmias in patients with impaired systolic function [21]. In a study conducted on 4172 patients diagnosed with acute heart failure, patients were divided into three groups based on their EF and GLS results. According to EF values, the groups were categorized as low (<40%), mildly to moderately reduced (40% to 49%), or preserved ejection fraction (≥50%). Based on GLS results, patients were grouped as having mild (>12.6%), moderate (8.1% < GLS < 12.5%), or severe (GLS ≤ 8.0%) strain reduction. During the 5-year follow-up period, the primary endpoint of this study was to determine all-cause mortality. It was found that the decrease in GLS had a stronger correlation with mortality than EF did. In the multivariable regression analysis of this study, each 1% increase in GLS was associated with a 5% reduction in mortality risk. At the conclusion of the study, the researchers argued that the GLS value is prognostically superior to EF [22]. In the literature, it has been reported that patients who experience malignant arrhythmic events after STEMI have lower GLS values compared to those who do not (−14.8 ± 4.7% versus −18.2 ± 3%) [23]. In another study conducted on heart failure patients, it was demonstrated that GLS possesses the highest prognostic value among echocardiographic parameters, including LVEF, in predicting major adverse cardiac events [24]. Consistent with the findings of all these studies in the literature, we believe that in our study, GLS may serve as a stronger prognostic indicator compared to LVEF in patients with impaired left ventricular systolic function following STEMI. In our study, the GLS value at discharge for Group 1 patients (with EF ≤ 35%) was significantly lower than that of Group 2 patients (with EF 36–49%) (−8.9 vs. −15.1, *p* < 0.001).

As a striking finding, it was observed that in group 1, patients with a GLS value below −9.55 did not achieve an LVEF greater than 35% during the 3-month follow-up, despite receiving optimal medical treatment. (LV GLS AUC (95% CI) = 0.814 (0.642–0.986), p: 0.005, sensitivity 75%, specificity 76.5%). Beyond the existing data in the literature showing that GLS has a better prognostic value than LVEF, we demonstrated in this study that the GLS measured at discharge could also predict low EF at the 3-month follow-up period.

Current cardiology guidelines recommend that for STEMI patients who have undergone revascularization and have an LVEF value of ≤35%, optimal medical treatment should be administered for 3 months before deciding on ICD implantation to prevent sudden cardiac death. If, after 3 months of treatment, the LVEF remains ≤35%, a decision is made regarding device implantation [3,4]. However, it is undeniable that these patients are at high risk for sudden cardiac death and ventricular arrhythmias during the interval before ICD implantation. In our study, patients with an EF below 35% and a GLS value below −9.55 at discharge did not show improvement in EF during the 3-month follow-up period, despite optimal medical treatment. Therefore, for these high-risk patients who do not yet meet the criteria for ICD according to current guideline recommendations but have a GLS value below −9.55, wearable ICD bridging therapy may be a viable treatment option to prevent adverse outcomes [3,25,26].

Additionally, there are suggestions in the literature that cardiac magnetic resonance imaging (MRI) can be used to identify high-risk groups [27]. A meta-analysis study showed that the assessment of myocardial fibrosis using LGE (late gadolinium enhancement) is a strong predictor of ventricular tachyarrhythmias (VTAs) in patients with ischemic and non-ischemic left ventricular dysfunction. This study highlights that the presence of myocardial fibrosis is an important factor that increases the risk of arrhythmias in these patients and that LGE could be a useful tool in the management of these patients [28]. In another study, the prognostic significance of global longitudinal strain (GLS) obtained through feature-tracking cardiac magnetic resonance (CMR) in patients with ST-Elevation Myocardial Infarction (STEMI) was investigated. The study examined the role of GLS in assessing left ventricular function post-STEMI and in predicting the long-term prognosis of patients. The findings indicate that GLS measurements could more accurately evaluate left ventricular function after STEMI, with low GLS values being associated with poor prognosis and higher mortality risk. Furthermore, GLS proved to be a more sensitive method than left ventricular ejection fraction (LVEF) in detecting subclinical myocardial dysfunction at an early stage. In conclusion, GLS could be an important tool for assessing cardiac function and predicting long-term prognosis in STEMI patients, and it may aid in treatment planning and risk stratification [29]. However, it is also a fact that cardiac magnetic resonance imaging is not accessible in every center for routine practice. Therefore, we believe that the measurement of left ventricular GLS by echocardiography, due to its ease of access and repeatability, could be useful in identifying high-risk patients for the prevention of sudden cardiac death, in addition to the EF value, when deciding on ICD implantation.

Due to its high clinical sensitivity and myocardial tissue specificity, troponin is a primary biomarker for the diagnosis of myocardial infarction (MI). It has been reported that the peak levels of troponin in STEMI patients are associated with infarct size, systolic dysfunction, and adverse outcomes [30,31,32]. Consistent with the literature, our study also found significantly higher peak troponin levels in the patient group with an EF value below 35%.

The most significant limitation of our study is that it is a single-center study with a relatively small number of patients. During the monitored follow-up period in the coronary intensive care unit, no life-threatening malignant arrhythmia attacks were observed in the included patients. However, the lack of rhythm Holter analyses, myocardial perfusion scintigraphy, or cardiac magnetic resonance imaging to assess scar presence/percentage during the hospital stay and the three-month post-discharge follow-up period can be considered a limitation. Since the primary aim of our study was to investigate whether left ventricular strain values at discharge could predict low EF at the third month, it does not provide data on long-term cardiac events. In accordance with our study hypothesis, only patients with impaired left ventricular systolic function, defined as an EF below 50%, were included. We believe that future studies, including patients with an EF of ≥50% post-MI, larger sample sizes, and longer follow-up periods in multicenter settings, could provide further insights. Therefore, in ischemic heart failure patients, the benefit of LV GLS assessment, in addition to LV EF and symptom status, for ICD decision making may be demonstrated with stronger sensitivity and specificity values.

## 5. Conclusions

In patients with ischemic heart disease and impaired systolic function, the LV GLS value obtained from strain echocardiography during the discharge period can predict LV EF values below 35% obtained from transthoracic echocardiography after three months. This finding may assist in the early identification of potential ICD candidates in this patient group, who are at high risk of life-threatening arrhythmic events due to the development of ischemic heart failure following STEMI, without the need to wait for three months.

## Figures and Tables

**Table 1:** Comparison of patients’ sociodemographic, hemodynamic, and laboratory characteristics.

Variable		Group 1	Group 2	*p*-Value
Gender *n* (%)	Female	3 (%20.6)	30 (%63.6)	0.22
	Male	26 (%78.4)	10 (%36.4)	
Age (Mean ± SD)		60.2 ± 9.5	54.7 ± 11	0.03
Height (cm)		171 (155–188)	170 (152–180)	0.99
Weight (kg)		78 (58–120)	80 (51–116)	0.53
Smoking Status *n* (%)	Never smoked	8 (%27.6)	9 (%22.5)	0.32
	Former smoker	7 (%24.1)	5 (%12.5)	
	Current smoker	14 (%48.3)	26 (%65)	
Hypertension *n* (%)		10 (34.5%)	15 (37.5%)	0.99
Hyperlipidemia *n* (%)		6 (20.7%)	1 (2.5%)	0.01
Diabetes Mellitus *n* (%)		8 (27.6%)	10 (25%)	0.8
History of MI *n* (%)		8 (27.6%)	5 (12.5%)	0.2
History of PCI (Balloon-Stent) *n* (%)		9 (31%)	5 (12.5%)	0.1
History of CABG *n* (%)		0 (0%)	2 (5%)	0.2
Peripheral Artery Disease *n* (%)		2 (6.9%)	1 (2.5%)	0.4
History of Stroke *n* (%)		1 (3.4%)	2 (5%)	0.75
Systolic BP (mmHg, Mean ± SD)		129.9 ± 22	135.2 ± 21.3	0.32
Diastolic BP (mmHg, Median + min–max)		80 (60–105)	80 (50–120)	0.74
Heart Rate (bpm, Mean + min–max)		85 (65–101)	79 (39–105)	0.11
Killip Classification *n* (%)	Class 1	23 (79.3%)	37 (92.5%)	0.18
	Class 2	5 (17.2%)	2 (5%)	
	Class 3	0 (0%)	1 (2.5%)	
	Class 4	1 (3.4%)	0 (0%)	
Time from Onset of Pain (hours)		5 (1–28)	4.5 (1–36)	0.19
Hemoglobin (g/dL)		14.4 (10.5–16.3)	14.6 (8.3–17.3)	0.54
CRP (mg/dL)		30 (1–286)	23.5 (2–163)	0.68
Glucose (mg/dL)		115 (81–336)	109.5 (81–285)	0.28
GFR (mL/min)		92 (57–120)	101.5 (37–120)	0.03
Creatinine (mg/dL)		0.8 (0.5–1.2)	0.8 (0.4–1.8)	0.08
LDL (mg/dL)		109 (29–168)	122.5 (35–290)	0.31
Admission Troponin (ng/L)		887 (1.5–50,000)	142 (1.4–50,000)	0.24
Peak Troponin (ng/L)		50,000 (389–50,000)	33,205.5 (642–50,000)	0.05

n: Number of patients, MI: Myocardial infarction, PCI: Percutaneous coronary intervention, CABG: Coronary artery bypass graft, SD: Standard deviation, BP: Blood pressure, CRP: C-reactive protein, GFR: Glomerular filtration rate, LDL: Low-density lipoprotein.

**Table 2:** Comparison of patients’ angiographic findings, discharge treatments, and 3-month follow-up outcomes.

Variable		Group 1	Group 2	*p*-Value
ECG Diagnosis *n* (%)	Anterior MI *n* (%)	26 (89.7%)	26 (65%)	0.55
	Inferior MI *n* (%)	3 (10.3%)	12 (30%)	
	Lateral MI *n* (%)	0 (0%)	2 (5%)	
Number of Affected Vessels		2 (1–3)	1 (1–3)	0.001
Door-to-Balloon Time (minutes)		31 (10–72)	29.5 (15–95)	0.75
Target Vessel for Intervention *n* (%)	LAD *n* (%)	26 (89.7%)	26 (65%)	0.06
	RCA *n* (%)	2 (6.9%)	11 (27.5%)	
	CX *n* (%)	1 (3.4%)	3 (7.5%)	
Procedure Success *n* (%)	Unsuccessful *n* (%)	0 (0%)	1 (2.5%)	0.4
	Successful *n* (%)	29 (100%)	39 (97.5%)	
Guideline-Directed Medical Therapy at Discharge	ASA *n* (%)	29 (100%)	40 (100%)	-
	P2Y12 Inhibitor *n* (%)	29 (100%)	40 (100%)	-
	Beta Blocker *n* (%)	29 (100%)	35 (87.5%)	0.05
	CCB *n* (%)	2 (6.9%)	11 (27.5%)	0.032
	ACEi/ARB *n* (%)	25 (86.2%)	34 (85%)	0.9
	ARNi *n* (%)	4 (13.8%)	2 (5%)	0.2
	MRA *n* (%)	22 (75.9%)	11 (22%)	<0.001
	SGLT-2 Inhibitor *n* (%)	8 (27.6%)	8 (20%)	0.65
	Diuretic *n* (%)	8 (27.6%)	4 (10%)	0.06
	Statin *n* (%)	29 (100%)	40 (100%)	-
3-Month Follow-up Outcome Data	Recurrent hospital admission	4 (13.8%)	3 (7.5%)	0.4
	Recurrent MI	2 (6.9%)	0 (0%)	0.1
	Recurrent intervention	13 (44.8%)	4 (10%)	0.001
	Heart-related death	1 (3.4%)	0 (0%)	0.42

MI: Myocardial infarction, LAD: Left anterior descending artery, RCA: Right coronary artery, CX: Circumflex artery, ASA: Acetylsalicylic acid, CCB: Calcium channel blocker, ACEi: Angiotensin-converting enzyme inhibitor, ARB: Angiotensin II receptor blocker, ARNi: Angiotensin receptor-neprilysin inhibitor, MRA: Mineralocorticoid receptor antagonist, SGLT-2: Sodium-glucose.

**Table 3:** Comparison of echocardiographic findings of patients.

Variable	Group 1 (Mean + Min–Max)	Group 2 (Mean + Min–Max)	*p*-Value
EF at Discharge (%)	32 (20–35)	44 (37–49)	<0.001
LV GLS	−8.9 (4.3–13.7)	−15.1 (11.4–20.3)	<0.001
EF on Day 90 (%)	35 (20–46)	53 (40–60)	<0.001
LVEDD (mm)	53 (46–61)	47 (42–54)	<0.001
LVESD (mm)	41 (32–54)	32 (30–44)	<0.001
IVS Thickness (mm)	10 (7–12)	11 (8–13)	0.02
PW Thickness (mm)	10 (6–11)	10 (8–12)	0.12
LA Diameter (mm)	41 (35–52)	37 (32–43)	<0.001
E/e′ Ratio	13.6 (7.3–21.7)	11.4 (5.7–17.7)	<0.001
RA Diameter (mm)	39 (30–48)	36 (31–42)	0.10
RV Diameter (mm)	33 (27–45)	31.5 (25–37)	0.11

LV GLS: Left Ventricular Global Longitudinal Strain, EF: Ejection Fraction, LVEDD: Left Ventricular End-Diastolic Diameter, LVESD: Left Ventricular End-Systolic Diameter, IVS: Interventricular Septum, PW: Posterior Wall, LA: Left Atrium, RA: Right Atrium, RV: Right Ventricle, E: Mitral early diastolic wave, e’: Mean early diastolic wave velocity at the septal and lateral mitral annulus.

**Table 4:** Univariate and multivariate regression analysis findings for predictors of EF ≤35% on day 90.

Variable	OR (95% CI)	*p*-Value	OR (95% CI)	*p*-Value
Age	0.979 (0.903–1.061)	0.60		
Gender	0 (0–0)	1.00		
Smoking	1.121 (0.4–3.14)	0.83		
Hypertension	0.476 (0.094–2.418)	0.37		
Hyperlipidemia	0.65 (0.098–4.29)	0.65		
Diabetes Mellitus	0.367 (0.06–2.252)	0.28		
Heart Failure	0 (0–0)	1.00		
History of MI	0.367 (0.06–2.252)	0.28		
MI Location	0 (0–0)	1.00		
Total Ischemic Time	0.984 (0.888–1.091)	0.76		
Door-to-Balloon Time	0.972 (0.914–1.033)	0.35		
Number of Diseased Vessels	0.922 (0.355–2.394)	0.87		
LV GLS	1.651 (1.134–2.405)	0.009	1.926 (1.024–2.625)	0.042
Peak Troponin Level	1.002 (0.985–1.050)	0.03	1.003 (0.990–1.069)	0.079

MI: Myocardial Infarction, PCI: Percutaneous Coronary Intervention, LV GLS: Left Ventricular Global Longitudinal Strain, CI: Confidence Interval.

## Data Availability

The datasets used and/or analyzed during this study are available from the corresponding author on reasonable request.

## References

[1] Demidova M.M., Carlson J., Erlinge D., Platonov P.G. (2015). Predictors of ventricular fibrillation at reperfusion in patients with acute ST-elevation myocardial infarction treated by primary percutaneous coronary intervention. Am. J. Cardiol..

[2] Epelde F. (2024). Impact of Exercise on Physiological, Biochemical, and Analytical Parameters in Patients with Heart Failure with Reduced Ejection Fraction. Medicina.

[3] Zeppenfeld K., Tfelt-Hansen J., de Riva M., Winkel B.G., Behr E.R., Blom N.A., Charron P., Corrado D., Dagres N., de Chillou C. (2022). 2022 ESC Guidelines for the management of patients with ventricular arrhythmias and the prevention of sudden cardiac death: Developed by the task force for the management of patients with ventricular arrhythmias and the prevention of sudden cardiac death of the European Society of Cardiology (ESC) Endorsed by the Association for European Paediatric and Congenital Cardiology (AEPC). Eur. Heart J..

[4] McDonagh T.A., Metra M., Adamo M., Gardner R.S., Baumbach A., Böhm M., Burri H., Butler J., Čelutkienė J., Chioncel O. (2021). 2021 ESC Guidelines for the diagnosis and treatment of acute and chronic heart failure: Developed by the Task Force for the diagnosis and treatment of acute and chronic heart failure of the European Society of Cardiology (ESC) with the special contribution of the Heart Failure Association (HFA) of the ESC. Eur. Heart J..

[5] Steinbeck G., Andresen D., Seidl K., Brachmann J., Hoffmann E., Wojciechowski D., Kornacewicz-Jach Z., Sredniawa B., Lupkovics G., Hofgärtner F. (2009). Defibrillator implantation early after myocardial infarction. N. Engl. J. Med..

[6] Hohnloser S.H., Kuck K.H., Dorian P., Roberts R.S., Hampton J.R., Hatala R., Fain E., Gent M., Connolly S.J. (2004). Prophylactic use of an implantable cardioverter–defibrillator after acute myocardial infarction. N. Engl. J. Med..

[7] Gronda E., Sacchi S., Benincasa G., Vanoli E., Napoli C. (2019). Unresolved issues in left ventricular postischemic remodeling and progression to heart failure. J. Cardiovasc. Med..

[8] Paul S. (2003). Ventricular remodeling. Crit. Care Nurs. Clin. N. Am..

[9] Dagres N., Hindricks G. (2013). Risk stratification after myocardial infarction: Is left ventricular ejection fraction enough to prevent sudden cardiac death?. Eur. Heart J..

[10] Sjøli B., Grenne B., Smiseth O.A., Edvardsen T., Brunvand H. (2011). The advantage of global strain compared to left ventricular ejection fraction to predict outcome after acute myocardial infarction. Echocardiography.

[11] Haugaa K.H., Smedsrud M.K., Steen T., Kongsgaard E., Loennechen J.P., Skjaerpe T., Voigt J.-U., Willems R., Smith G., Smiseth O.A. (2010). Mechanical dispersion assessed by myocardial strain in patients after myocardial infarction for risk prediction of ventricular arrhythmia. JACC Cardiovasc. Imaging.

[12] Lang R.M., Badano L.P., Mor-Avi V., Afilalo J., Armstrong A., Ernande L., Flachskampf F.A., Foster E., Goldstein S.A., Kuznetsova T. (2015). Recommendations for cardiac chamber quantification by echocardiography in adults: An update from the American Society of Echocardiography and the European Association of Cardiovascular Imaging. Eur. Heart J.-Cardiovasc. Imaging.

[13] Collier P., Phelan D., Klein A. (2017). A test in context: Myocardial strain measured by speckle-tracking echocardiography. J. Am. Coll. Cardiol..

[14] Bansal M., Kasliwal R.R. (2013). How do I do it? Speckle-tracking echocardiography. Indian Heart J..

[15] Klaeboe L.G., Edvardsen T. (2019). Echocardiographic assessment of left ventricular systolic function. J. Echocardiogr..

[16] Luis S.A., Chan J., Pellikka P.A. (2019). Echocardiographic Assessment of Left Ventricular Systolic Function: An Overview of Contemporary Techniques, Including Speckle-Tracking Echocardiography.

[17] Emond M., Mock M.B., Davis K.B., Fisher L.D., Holmes D.R., Chaitman B.R., Kaiser G.C., Alderman E., Killip T. (1994). Long-term survival of medically treated patients in the Coronary Artery Surgery Study (CASS) Registry. Circulation.

[18] Curtis J.P., Sokol S.I., Wang Y., Rathore S.S., Ko D.T., Jadbabaie F., Portnay E.L., Marshalko S.J., Radford M.J., Krumholz H.M. (2003). The association of left ventricular ejection fraction, mortality, and cause of death in stable outpatients with heart failure. J. Am. Coll. Cardiol..

[19] Stanton T., Leano R., Marwick T.H. (2009). Prediction of all-cause mortality from global longitudinal speckle strain: Comparison with ejection fraction and wall motion scoring. Circ. Cardiovasc. Imaging.

[20] Potter E., Marwick T.H. (2018). Assessment of left ventricular function by echocardiography: The case for routinely adding global longitudinal strain to ejection fraction. JACC Cardiovasc. Imaging.

[21] Nikoo M.H., Naeemi R., Moaref A., Attar A. (2020). Global longitudinal strain for prediction of ventricular arrhythmia in patients with heart failure. ESC Heart Fail..

[22] Park J.J., Park J.-B., Park J.-H., Cho G.-Y. (2018). Global longitudinal strain to predict mortality in patients with acute heart failure. J. Am. Coll. Cardiol..

[23] Haugaa K.H., Grenne B.L., Eek C.H., Ersbøll M., Valeur N., Svendsen J.H., Florian A., Sjøli B., Brunvand H., Køber L. (2013). Strain echocardiography improves risk prediction of ventricular arrhythmias after myocardial infarction. JACC Cardiovasc. Imaging.

[24] Mignot A., Donal E., Zaroui A., Reant P., Salem A., Hamon C., Monzy S., Roudaut R., Habib G., Lafitte S. (2010). Global longitudinal strain as a major predictor of cardiac events in patients with depressed left ventricular function: A multicenter study. J. Am. Soc. Echocardiogr..

[25] Cheung C.C., Olgin J.E., Lee B.K. (2021). Wearable cardioverter-defibrillators: A review of evidence and indications. Trends Cardiovasc. Med..

[26] Zoppo F., Mugnai G., Lupo A., Zerbo F. (2020). Bridge to avoid ICD implantation with wearable cardioverter defibrillators. Adv. Interv. Cardiol./Postępy Kardiol. Interwencyjnej.

[27] Halliday B.P., Gulati A., Ali A., Guha K., Newsome S., Arzanauskaite M., Vassiliou V.S., Lota A., Izgi C., Tayal U. (2017). Association Between Midwall Late Gadolinium Enhancement and Sudden Cardiac Death in Patients with Dilated Cardiomyopathy and Mild and Moderate Left Ventricular Systolic Dysfunction. Circulation.

[28] Disertori M., Rigoni M., Pace N., Casolo G., Masè M., Gonzini L., Lucci D., Nollo G., Ravelli F. (2016). Myocardial Fibrosis Assessment by LGE Is a Powerful Predictor of Ventricular Tachyarrhythmias in Ischemic and Nonischemic LV Dysfunction: A Meta-Analysis. JACC Cardiovasc. Imaging.

[29] Reindl M., Tiller C., Holzknecht M., Lechner I., Beck A., Plappert D., Gorzala M., Pamminger M., Mayr A., Klug G. (2019). Prognostic Implications of Global Longitudinal Strain by Feature-Tracking Cardiac Magnetic Resonance in ST-Elevation Myocardial Infarction. Circ. Cardiovasc. Imaging.

[30] Byrne R.A., Rossello X., Coughlan J.J., Barbato E., Berry C., Chieffo A., Claeys M.J., Dan G.-A., Dweck M.R., Galbraith M. (2024). 2023 ESC guidelines for the management of acute coronary syndromes: Developed by the task force on the management of acute coronary syndromes of the European Society of Cardiology (ESC). Eur. Heart J. Acute Cardiovasc. Care.

[31] Hallén J. (2012). Troponin for the estimation of infarct size: What have we learned?. Cardiology.

[32] Khullar N., Buckley A.J., O’connor C., Ibrahim A., Ibrahim A., Ahern C., Cahill C., Arnous S., Kiernan T.J. (2022). Peak troponin T in STEMI: A predictor of all-cause mortality and left ventricular function. Open Heart.

